# A new efficiency calibration methodology for different atmospheric filter geometries by using coaxial Ge detectors

**DOI:** 10.1007/s11869-023-01336-x

**Published:** 2023-03-10

**Authors:** A. Barba-Lobo, J. P. Bolívar

**Affiliations:** Radiation Physics and Environment Group (FRYMA), Department of Integrated Sciences, Center for Natural Resources, Health and Environment (RENSMA), University of Huelva, 21071 Huelva, Spain

**Keywords:** Atmospheric filters, Particulate matter, Atmospheric aerosols, Efficiency calibration, Gamma-ray spectrometry, Ge detectors

## Abstract

The study of the different pollutants present in atmospheric aerosols such as trace elements and radionuclides is essential to assess the air quality. To analyze the particulate matter (PM), atmospheric filters with different dimensions and geometries (rectangular, circular, slotted, and square filters) are usually employed. Regarding the pollutants existing in atmospheric aerosols, radionuclides are usually analyzed due to their multiple applications such as either in the environmental radiological control or as tracers of atmospheric processes. Therefore, this study aims to develop a new and general methodology to calibrate in efficiency coaxial Ge detectors to properly determine radionuclides present in the PM by gamma-ray spectrometry for several filter types. For this, granular certified reference materials (CRM) containing only natural radionuclides (^238^U-series, ^232^Th-series, and ^40^ K) were selected. Several granular solid CRMs were chosen allowing us to reproduce the same PM deposition geometry and to assure the homogeneity of the added CRMs. These are the main advantages in relation to the typical methods that use liquid CRMs. Furthermore, for filters whose surfaces are relatively large, they were cut in several pieces and placed one on top of the other, achieving the same geometry than the PM deposited onto the filter. Then, the experimental full-energy peak efficiencies (*FEPEs*) were obtained for each energy of interest (*E*_*γ*_) and they were fitted versus *E*_*γ*_, finding a general *FEPE* function for each filter type. Finally, this methodology was validated for both natural and artificial radionuclides (from 46 to 1332 keV) by using different filter types employed in proficiency test exercises, obtaining |*z*_score_|< 2 for all cases.

**Supplementary Information:**

The online version contains supplementary material available at 10.1007/s11869-023-01336-x.

## Introduction

In the last decades, the research of the air quality and its health impacts are essential topics within the environmental and atmospheric sciences. For this, the particulate matter (PM) needs to be studied in depth since it can provide a very valuable information about the pollution levels present in the atmospheric aerosols. In addition, the assessment of the pollution associated to the atmospheric aerosols can be employed in order to get a better comprehension about the different factors involved in the global climate (Davidson et al. 2005; Fuzzi et al. 2015; Lu et al. 2016; Ouyang 2013), as well as about the evolution of certain diseases such as the ones caused by the SARS-CoV-2 virus (Mehmood et al. 2021a; Mehmood et al. 2021b; Paez-Osuna et al. 2022).

The atmospheric PM is collected onto filters with different geometry and dimensions depending on the objective of the study, where the PM_10_, PM_2.5_, and PM_1.0_ are the most used samplers (Barba-Lobo et al. 2022; Dueñas et al. 2009; Ordúz 2012). Furthermore, there is another type of sampler, the cascade impactor (CI), that is very useful since it allows us to classify the particulate matter present in the atmospheric aerosols as a function of the aerodynamic diameter (*ad*) of the particles (Aba et al. 2020; Kwon et al. 2003). This sampler can provide us information about the affinity existing between the different pollutants present in the atmospheric aerosols and the size of the aerosol particles. In the cases of the PM_10_, the air flow needs to be fixed at 68 m^3^ h^−1^ following the USEPA Compendium Method IO-2.1 (EPA 1999), while for PM_2.5_ and PM_1.0_ samplers, the air flow is fixed at 30 m^3^ h^−1^ according to the European Standard UNE-EN 14,907 (EN 14,907, 2005). In order to be able to carry out samplings spending a sampling duration as short as possible, it is recommendable to make use of a very high-volume air aerosol sampler, model ASS-500 (Valkovic 2000). This sampler allows us to reach air flows ranged about 500 m^3^ h^−1^ and 600 m^3^ h^−1^. Furthermore, this last type of sampler is very recommendable to reduce the minimum detectable activity concentration (*mda*), since it is inversely proportional to the air flow, which makes possible to get *mda* values about 1 μBq m^−3^.

Among the different types of pollutants present in atmospheric aerosols, radionuclides can be found. They can be employed for multiple purposes such as environmental radiological control, obtention of information about the origin of a certain contaminant source, calculation of the residence times of atmospheric aerosols and of the external and internal dose rates, and the trace of air masses and atmospheric processes (Abdo et al. 2021; Baskaran and Shaw 2001; Papastefanou and Bondietti 1991; Renoux 1987; Srinivas et al. 2014). For this, there are several radionuclides such as ^7^Be, ^210^Po, ^214, 212, 210^Pb, and ^214, 212, 210^Bi that are usually employed in the atmospheric studies (Dalaka et al. 2019; Papastefanou and Ioannidou 1996; Poet et al. 1972; Tokieda et al. 1996). These radionuclides can also allow us to find atmospheric deposition patterns for different regions of interest considering a wide variety of meteorological conditions (Baskaran and Swarzenski 2007; Lozano et al. 2011). Furthermore, in the case of the short-lived progenies of the ^222^Rn (radon) and ^220^Rn (thoron), that is, the ^214^Pb and ^214^Bi, and ^212^Pb and ^212^Bi, respectively, they are also useful to determine equilibrium factors (Barba-Lobo et al. 2023; Chalupnik et al. 2021) which are related to the internal dose rate.

To determine the activity concentrations of radionuclides in the atmospheric aerosols, a calibration in efficiency of the selected detector is needed. For this, this study aims to develop a new and general methodology based on the use of granular solid CRMs for the efficiency calibration of coaxial Ge detectors, which are widely employed for the determination of gamma-ray emitters by using numerous atmospheric filter types.

## Materials and methods

### Materials

In this study, several types of atmospheric filters were selected to develop the new methodology for the efficiency calibration, considering the great majority of the filter types employed by laboratories when analyzing the particulate matter. The filters selected in our case were quartz fiber filters characterized by having different geometries and dimensions: rectangular filters (20 × 25 cm^2^), circular filters (diameters = 47 mm, 150 mm), slotted filters (which are employed for the CI samplers, 13.6 × 14.3 cm^2^), and square polypropylene filters (44 × 44 cm^2^), which are used in the case of the very high-volume samplers, as the ASS-500 sampling station. Let us call these filters as R, C-47, C-150, SL, and SQ, respectively, to easily be identified.

For the determination of the gamma-ray emitters, an extended energy range (XtRa) high purity germanium (HPGe) detector (model GX3519 provided by Canberra) was chosen. The XtRa detector has a relative efficiency of 38.4% at 1332 keV (^60^Co) in relation to a 3″ $\times$ 3″ NaI(Tl) detector, a full width at half maximum (FWHM) of 1.74 keV and 0.88 keV at 1332 keV and 122 keV, respectively, and a peak-to-Compton ratio of 67.5:1. The XtRa detector is connected to a conventional electronic chain, which is composed on a preamplifier, an amplifier, an analog-to-digital converter, and a multichannel analyzer, where the software employed for the acquisition and analysis of the gamma spectra was Genie 2000 (Canberra Industries 2004).

In order to carry out the efficiency calibration of the XtRa detector, standards provided by the International Atomic Energy Agency (IAEA), RGU-1, RGTh-1, and RGK-1, were used, which are certified reference materials (CRM) and contain natural radionuclides belonging to the ^238^U-series, ^232^Th-series, and ^40^ K, whose certified activity concentrations were 4940(15) Bq kg^−1^, 3250(45) Bq kg^−1^, and 14,000(200) Bq kg^−1^, respectively, where the uncertainties are given at one sigma confidence level (IAEA 1987).

For the validation of the methodology developed in this study, atmospheric filters selected to carry out the inter-comparison exercises in 2017 and 2021, which were organized by the CSN and CIEMAT, were employed, where the acronyms CSN and CIEMAT are referred to the Spanish Nuclear Safety Council (“Consejo de Seguridad Nuclear”) and the Centre for Energy, Environment and Technology Research (“Centro de Investigaciones Energéticas, Medioambientales y Tecnológicas”), respectively.

### Method

**Fig. 1:**
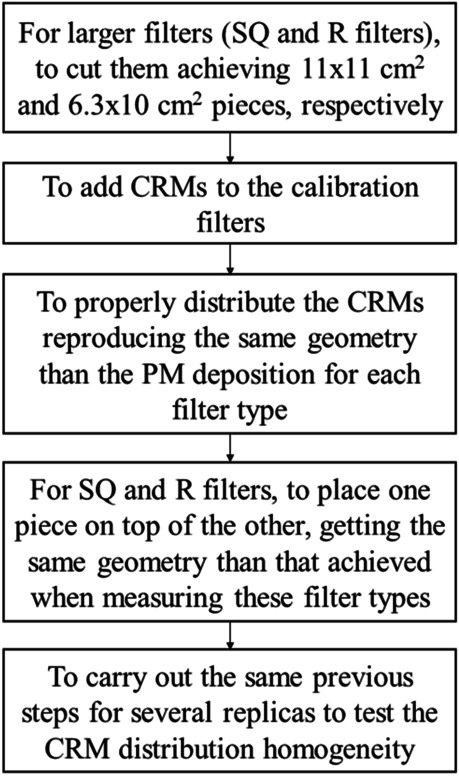
Scheme of the experimental procedure for the preparation of the different filter types (SL, C-150, C-47, R, and SQ filters) to carry out the efficiency calibrations

To carry out the efficiency calibration of the detector by using the quartz fiber filters (R, C-47, C-150, and SL), some amount of RGU-1 was added and very well extended onto each filter type (see Fig. 1), achieving the homogeneity of this certified material throughout all the filter surfaces. In the case of the SL filters, the calibration was carried out using two filters, where 0.5115(2) g and 0.5014(2) g are the RGU-1 amounts added to each filter. In the case of the C-47 filters, three filters were used, where the added masses were 0.4953(2) g, 0.4941(2) g and 0.5009(2) g, respectively, while for C-150 filters, three filters were also chosen, where the added masses were 0.5029(2) g, 0.4898(2) g, and 0.5006(2) g, respectively. Then, for the R filters, three filters were used for the calibration, where the added masses were 0.6018(2) g, 0.5913(2) g, and 0.5914(2) g, respectively. In the case of the R filters, these filters were cut in several rectangular pieces which were placed one piece on top of the other, getting the same geometry than the one achieved when measuring this filter type (6.3 × 10 cm^2^) (see Fig. 1). Then, the RGU-1 standard is spread for each one of these pieces, covering the same regions than the ones where the particulate matter is deposited onto the R filters during the samplings. When proceeding in this way for the R filters, it is possible to make it easier to get the homogeneity of the RGU-1 standard when adding onto this filter type, as well as to get reproducing the distribution of the particulate matter when depositing onto the problem filters.

In the case of the SQ filters, RGU-1, RGTh-1, and RGK-1 standards were selected for the efficiency calibration. The three standards were well mixed among them, and three filters were employed, where the amount of the added mixtures were 2.5011(2) g, 2.5017(2) g, and 2.4906(2) g, respectively, where the amount of each standard was one-third of the mass of each mixture added. Analogously to the case corresponding to the R filters, the SQ filters were also cut in pieces where each one had the same dimensions than the any problem filter ones after being folded to be measured (11 × 11 cm^2^) (see Fig. 1). Furthermore, the mixture of the three standards was spiked to each piece, covering the same regions than the ones covered when depositing the particulate matter onto the problem filters.

By using this methodology developed in this study, a more proper efficiency calibration is achieved in comparison with the typically used methods, where certified liquid samples are added to the filters by using a pipette (Idoeta et al. 2021; López-López et al. 2020; Tourang et al. 2021). The usage of certified liquid samples makes it easier the existence of inhomogeneities, and it is more likely that the concentrations of the radionuclides present in these standards can be altered since they are dissolutions. Moreover, since the particulate matter is a solid sample, it is more recommendable to calibrate in efficiency by using solid samples than liquid ones. This allows us to reproduce the same distribution of the particulate matter when depositing onto the filters, as well as to obtain similar self-absorption effects for the emitted photons in the cases of the calibration and problem filters. In the case of the bigger filters (R and SQ filters), these filters were cut in several pieces following the same geometry and dimensions than the ones obtained when measuring the problem filters. This is another reason why this methodology is more proper than the typical methods which employ the calibration filters without cutting them (Idoeta et al. 2021; López-López et al. 2020; Tourang et al. 2021). This makes it more difficult to achieve the homogeneity of the spiked standard, not reproducing the distribution of the particulate matter deposited onto the problem filters in a proper way.

To determine natural and artificial radionuclides by gamma-ray spectrometry, nowadays, the detector calibrations by Monte Carlo simulations have become generalized, even in the case of using atmospheric filters (Aba et al. 2020). There are many cases for which detectors are not characterized because of being relatively old. To proceed with it, high costs for the characterization and the new Monte Carlo software are involved. During the characterization procedure, the detectors must be outside the laboratory, and consequently, inoperative for several months. In contrast to Monte Carlo simulations, in this study, an experimental methodology has been developed by which it is possible to calibrate coaxial detectors relatively quickly for atmospheric filters using calibration standards, which do not imply so high costs.

Therefore, this is the first study to address in depth the experimental calibration of coaxial Ge detectors for atmospheric filters by using granular solid CRMs, developing an exhaustive calibration filter preparation method to reproduce the particulate matter deposition geometry with a high accuracy.

To carry out these calibrations, the experimental *FEPEs*, ${\varepsilon }_{\mathrm{exp}}$, were calculated by using the following equation:

1 $${\varepsilon }_{\mathrm{exp}} \left({E}_{\gamma }\right)=\frac{G-B-F-I}{{P}_{\gamma } {a}_{c} {m}_{c} t}=\frac{N}{{P}_{\gamma } {a}_{c} {m}_{c} t}$$

where *G*, *B*, *F*, *I*, and *N* are the total number (gross) of counts, the Compton continuum, the background due to environmental conditions existing in the laboratory, the interference term, and the net counts, respectively, where all of them are referred to the full-energy peak of interest. Then, ${P}_{\gamma }$ is the probability of gamma emission (taken from DDEP 2017); ${a}_{c}$ and ${m}_{c}$ are the activity concentration and the mass added to each filter of the calibration standard, respectively, which have been used in the calibration procedure. Then, ${E}_{\gamma }$ are the selected gamma emission energies and *t* is the measurement time. Furthermore, it has been considered that *I* ~ 0 for all selected ${E}_{\gamma }$.

After the counting of the filters by using the XtRa detector, the experimental efficiencies were calculated for each filter type. For this, the full-energy peak efficiency (*FEPE*) was calculated for each selected gamma emission energy (${E}_{\gamma }$). Afterwards, an average *FEPE* value was calculated for each energy in the case of each sampler type. Finally, the logarithms of the experimental *FEPEs* were fitted versus $\mathrm{ln}({E}_{\gamma }/{E}_{0})$, where ${E}_{0}$ = 1 keV, achieving to obtain a general efficiency function for each filter type.

## Results and discussion

### Calibration in efficiency for each filter type

In Tables S.1, S.3, S.5, S.7, and S.9 (see Supplementary Material), the ${\varepsilon }_{\mathrm{exp}}^{i (j)}$ values obtained in the cases of the SL, C-150, C-47, R, and SQ filters were shown, respectively, for each filter chosen to accomplish the calibrations in efficiency, where *i* = SL, C-150, C-47, R, and SQ, and *j* = 1, 2, and 3 is the number of the filter used for each type of geometry. Furthermore, in Tables S.1, S.3, S.5, S.7, and S.9, the average experimental efficiencies, ${\varepsilon }_{\mathrm{exp}}^{i}$, have been provided for each filter type.

**Fig. 2:**
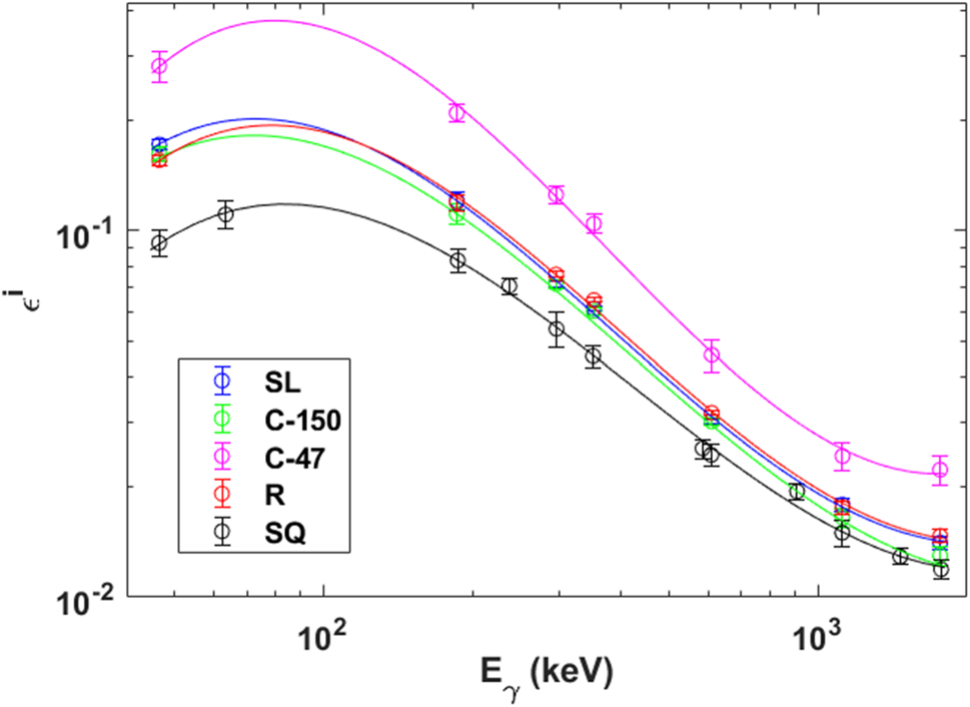
Experimental efficiencies resulting from the efficiency calibration carried out for five types of filters: slotted filters (SL), circular filers whose diameters are 47 mm and 150 mm (C-47 and C-150, respectively), rectangular filters (R), and squared filters (SQ). Besides, the fittings accomplished for the experimental efficiencies, ${\varepsilon }_{\mathrm{exp}}^{i}$, versus the gamma emission energy, ${E}_{\gamma }$, where *i* = SL, C-150, C-47, R, and SQ, have also been shown

The average of the full energy peak efficiency, ${\varepsilon }_{\mathrm{exp}}^{i}$, was plotted versus ${E}_{\gamma }$ for each filter type by using logarithmic scales for both axes of the graph (Fig. 2). As can be seen in this figure, the behavior of ${\varepsilon }_{\mathrm{exp}}^{i}$ versus ${E}_{\gamma }$, by using logarithmic scales for both variables, is clearly polynomial type. Consequently, the following function was chosen in order to fit $\mathrm{ln}({\varepsilon }_{\mathrm{exp}}^{i})$ versus $\mathrm{ln}({E}_{\gamma }/{E}_{0})$ (Bolívar et al. 1996; El-Daoushy and García-Tenorio 1995; Gilmore and Hemingway 1995; Martínez-Ruiz et al. 2007):

2 $$\mathrm{ln}\left({\varepsilon }_{\mathrm{exp}}^{i}\right)\left({E}_{\gamma }\right)=\sum_{k=0}^{3}{a}_{k}^{i} \mathrm{ln}{\left(\frac{{E}_{\gamma }}{{E}_{0}}\right)}^{k}$$

where ${a}_{k}^{i}$ are the parameters resulting from the fits, *k* = 0, 1, 2, and 3, and *I* = SL, C-150, C-47, R, and SQ. Then, ${E}_{\gamma }$ is the gamma emission energy of interest and ${E}_{0}$ = 1 keV. It is necessary to clarify that the ${E}_{\gamma }$, which were selected to carry out the calibrations (see Tables S.1, S.3, S.5, S.7 and S.9), were chosen because of having the highest ${P}_{\gamma }$ values corresponding to the radionuclides belonging to the ^238^U-series, ^232^Th-series, and ^40^ K, achieving to obtain high counting rates and, therefore, to accomplish these calibrations relatively quickly.

Moreover, note that the highest *FEPEs* were obtained for the C-47 filter. This is consistent since this filter type has a diameter close to the XtRa detector one (58.5 mm), which minimizes the number of photons not detected. On the contrary, for the SQ filter, its size is much larger than the detector window, which causes a significant increase of non-detected photons and, therefore, a decrease of the *FEPEs*. In the case of the SL, C-150, and R filters, it is possible to observe that the *FEPEs* were similar from each other which is due to the similar size of the filters after being folded.

In Tables S.2, S.4, S.6, S.8, and S.10 (see Supplementary Material), the ${a}_{k}^{i}$ parameters can be found for the SL, C-150, C-47, R, and SQ filters, respectively. Furthermore, in Tables S.1, S.3, S.5, S.7, and S.9, the relative residuals, *Res* (%), resulting from the fits provided by Eq. 2 can be found for each selected energy in the cases of SL, C-150, C-47, R, and SQ filters, respectively, where the Res values were calculated by using the following equation:

3 $$Res ({\%})=100\left(\frac{{y}_{\mathrm{fit}}^{\left({E}_{\gamma }\right)}}{{y}_{\mathrm{exp}}^{\left({E}_{\gamma }\right)}}-1\right)$$

where ${y}_{\mathrm{exp}}^{\left(i\right)}$ and ${y}_{\mathrm{fit}}^{\left(i\right)}$ are the values obtained experimentally and the ones obtained by a fitting function for a magnitude *y*, respectively, for each energy ${E}_{\gamma }$.

**Fig. 3:**
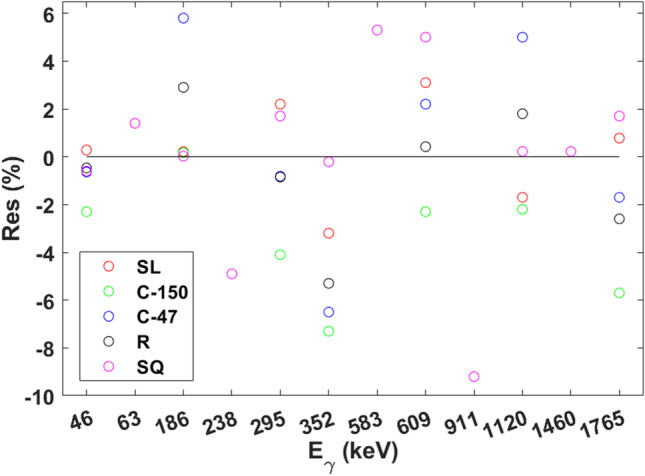
Relative residuals, *Res* (%), resulting from fitting the experimental *FEPEs* versus the gamma emission energy, ${E}_{\gamma }$, by using the general efficiency function found for each filter type (SL, C-150, C-47, R, and SQ)

As can be seen in Fig. 3, all |*Res*| values were less than 9.2, but it is easy to realize that the great majority of the |*Res*| values were less than 3 and, consequently, the fits provided by Eq. 2 agreed very well with the ${\varepsilon }_{\mathrm{exp}}^{i}$ values for the five filter types (see Tables S.1, S.3, S.5, S.7, and S.9 in order to check numerically the values obtained for *Res*). Furthermore, in Tables S.2, S.4, S.6, S.8, and S.10, it is possible to find the ${R}^{2}$ values resulting from each fitting, where ${R}^{2}$ were 0.9995, 0.9993, 0.998, 0.9991, and 0.998 in the cases of the SL, C-150, C-47, R, and SQ filters, respectively, which further corroborate the great fits provided by Eq. 2.

In addition, note that the *Res* values obtained at each energy for each filter type were well distributed between the positive and negative panels. This allows us to prove that the *Res* values do not follow any systematic tendency.

### Validation of the efficiency calibration methodology

In this section, the validations of the methodology developed in this study are shown. For this, filters of the C-47, R, and SQ types were employed, which were selected for the Inter-comparison exercises organized by the CSN and CIEMAT in 2017 and 2021. It is necessary to clarify that the filters of the C-150 and SL types are unusually employed in the inter-comparison exercises, and for this reason, they were not included in this section. However, they have also been included in this study due to the great importance that these filter types have for a proper and complete analysis of the particulate matter present in atmospheric aerosols. Furthermore, considering the very low values obtained for *Res* in the case of the C-150 and SL filters when comparing the resulting experimental *FEPEs* for each filter type replica, it is possible to test the very good interval validation achieved at each selected ${E}_{\gamma }$.

In order to proceed with these validations, it was necessary to make use of the *z*_score_, which can be obtained by the following equation:

4 $${z}_{\mathrm{score}} =\frac{{a}_{i} - \mathrm{med}({a}_{i})}{\sigma \left(\mathrm{med}\left({a}_{i}\right)\right)}$$

where ${a}_{i}$ is the activity concentration value obtained for each radionuclide by using this methodology, $\mathrm{med}({a}_{i})$ is the median value of the activity concentration for each radionuclide resulting from considering the values provided by all the laboratories that participate in the Inter-comparison exercise, and $\sigma \left(\mathrm{med}\left({a}_{i}\right)\right)$ is the absolute deviation of the $med({a}_{i})$.

In Tables 1, 2 and 3, the validations carried out by using the filters C-47, R, and SQ, respectively, were shown, where both natural and artificial radionuclides (^214, 210^Pb, ^214^Bi and ^226^Ra, and ^54^Mn, ^60, 57^Co, ^65^Zn, ^137, 134^Cs and ^241^Am, respectively) were selected in order to achieve more complete validations.

**Table 1:** Validation of the efficiency calibration carried out for the circular filters whose diameter is 47 mm (C–47). For this, a filter employed in an inter-comparison exercise organized by the CSN and the CIEMAT in 2017 was selected

RN	${E}_{\gamma }$(keV)	*a* (Bq filter^−1^)	$\mathrm{med}\left(a\right)$(Bq filter^−1^)	*z*_score_
^226^Ra	185.96	0.88 (8)	0.50 (10)	2.0
^214^Pb	351.93	0.47 (2)	0.50 (4)	− 0.3
^214^Bi	609.31	0.49 (4)	0.48 (5)	0.1
^210^Pb	46.54	1.70 (4)	1.77 (15)	− 0.2
^54^Mn	834.84	1.06 (2)	1.10 (12)	− 0.3
^57^Co	122.10	0.388 (8)	0.39 (4)	0.0
^60^Co	1332.50	0.720 (15)	0.72 (3)	0.0
^134^Cs	604.70	0.60 (8)	0.62 (4)	− 0.2
^137^Cs	661.80	0.469 (9)	0.43 (3)	0.6
^241^Am	59.54	0.207 (6)	0.210 (15)	− 0.1

**Table 2:** Validation of the efficiency calibration carried out for the rectangular filters (R). For this, a filter employed in an inter-comparison exercise organized by the CSN and the CIEMAT in 2017 was selected

RN	${E}_{\gamma }$(keV)	*a* (Bq filter^−1^)	$\mathrm{med}\left(a\right)$(Bq filter^−1^)	*z*_score_
^226^Ra	185.96	1.2 (2)	1.00 (2)	0.5
^54^Mn	834.84	1.15 (12)	0.801 (13)	1.5
^60^Co	1332.50	0.85 (9)	0.704 (12)	0.9
^134^Cs	604.70	0.75 (8)	0.620 (13)	0.9
^137^Cs	661.80	0.60 (7)	0.484 (9)	0.9

**Table 3:** Validation of the efficiency calibration carried out for the squared filters (SQ). For this, a filter employed in an inter-comparison exercise organized by the CSN and the CIEMAT in 2021 was selected

RN	${E}_{\gamma }$(keV)	*a* (Bq filter^−1^)	$\mathrm{med}\left(a\right)$(Bq filter^−1^)	*z*_score_
^226^Ra	185.96	1.7 (2)	2.00 (8)	− 1.1
^54^Mn	834.84	1.06 (2)	1.00 (4)	0.4
^57^Co	122.10	0.173 (15)	0.203 (8)	− 1.0
^60^Co	1332.50	1.94 (3)	2.06 (8)	− 0.4
^65^Zn	1115.55	1.95 (13)	1.84 (8)	0.4
^134^Cs	801.93	0.33 (3)	0.350 (14)	0.4
^137^Cs	661.80	0.433 (15)	0.412 (17)	0.3
^241^Am	59.54	0.264 (11)	0.352 (15)	− 1.3

As can be seen in Tables 1, 2, and 3, the |*z*_score_| values obtained for each chosen artificial and natural radionuclide agreed well with the median obtained in the proficiency tests, accomplishing that all of them were less than 2. This clearly demonstrates the very good validation of the methodology proposed in this study, for both artificial and natural radionuclides as well as for both low and high energies ($\le$ 150 keV and > 150 keV, respectively). Furthermore, making use of this satisfactory validation, it is also possible to conclude that the homogeneity accomplished during the preparation of the calibration filters was very proper. This allows us to consider it as a very recommendable methodology for the efficiency calibration of coaxial Ge detectors by using a very wide variety of filter types. Furthermore, this methodology can be applied in a very proper way for the determination of natural and artificial radionuclides whose gamma emission energies are distributed throughout all the energy range of interest (from 46 to 1332 keV).

## Conclusions

In this study, an original methodology has been developed for the full-energy peak efficiency (*FEPE*) calibration of coaxial Ge detectors by using a great variety of atmospheric filters. These filters are characterized by having different geometries and dimensions (rectangular, circular, square, and slotted filters), which are usually used when carrying out research related to the particulate matter (PM) present in atmospheric aerosols.

To prepare the calibration samples (filters), granular certified reference materials were selected, and they were well spread throughout the same regions than the ones covered by the PM when depositing onto the problem filters. This allows us to achieve a very high homogeneity of the calibration standard material added, reproducing the same distribution than the one followed by the PM onto the filters. Furthermore, because of the significant dimensions in the cases of the rectangular and square filters, it was necessary to cut them in several pieces which were placed one piece on top of the other. This was carried out following the same geometry and dimensions than the ones obtained when measuring the problem filters, where the calibration standards were spiked onto each one of these pieces. This makes it easier to achieve the homogeneity of the certified material spread and to reproduce the PM distribution onto the filter surface for filters whose surfaces are relatively large.

After preparing the calibration filter, the different filter types were counted by gamma-ray spectrometry, obtaining the experimental *FEPEs* for each filter and each selected energy. Afterwards, the experimental *FEPEs* were fitted by using a polynomial function, allowing us to find a general *FEPE* function for each filter type covering a very wide gamma emission energy range (from 46 to 1765 keV). The relative average residual and *R*^2^ values resulting from these fits were very good, which were 1.6%, 3.4%, 3.2%, 2.1%, and 2.5%, and 0.9995, 0.9993, 0.998, 0.9991, and 0.998, respectively, for the SL, C-150, C-47, R, and SQ filters, respectively. This proves the good agreement between the experimental *FEPEs* and the values provided by the semiempirical general function found for the *FEPE*.

Finally, the methodology developed in this study was subjected to several validation tests by using filters employed in inter-comparison exercises. A very good validation was achieved for both natural and artificial radionuclides whose gamma emission energies are well distributed throughout the energy range of interest, obtaining |*z*_score_| values less than 2 for all cases. This allows us to conclude that this methodology is very recommendable in order to determine natural and artificial radionuclides, that are present in atmospheric aerosols, for a very wide energy range and variety of filter types.

## Supplementary Information

Below is the link to the electronic supplementary material.

Supplementary file1 (DOCX 49 KB)

## Data Availability

All data and materials as well as software application or custom code support their published claims and comply with field standards.
