# Supplementary material for: A new efficiency calibration methodology for different atmospheric filter geometries by using coaxial Ge detectors
Source: Air Qual Atmos Health. 2023 Mar 10;16(6):1207–14. doi: 10.1007/s11869-023-01336-x (PMC10000343; doi:10.1007/s11869-023-01336-x)
Supplement: Supplementary file 1 — Supplementary file1 (DOCX 49 KB) [file 11869_2023_1336_MOESM1_ESM.docx]

Appendix A. Supplementary Material

| **Table S.1**  Experimental efficiencies obtained in the calibration procedure for slotted filters (SL), $\varepsilon_{exp}^{SL (j)}$, where j = 1, 2. Then, $m_{RGU-1}^{SL (j)}$ is the amount of RGU-1 added to each filter “j” in order to carry out the efficiency calibration. By using the $\varepsilon_{exp}^{SL (j)}$ values obtained for both filters, an average experimental efficiency was calculated, $\varepsilon_{exp}^{SL}$, for each $E_{\gamma}$, where $\sigma\left( \varepsilon_{exp}^{SL \left( j \right)} \right)$ and $\sigma\left( \varepsilon_{exp}^{SL} \right)$ are the uncertainties related to $\varepsilon_{exp}^{SL (j)}$ and $\varepsilon_{exp}^{SL}$, respectively, and *Res* (%) are the relative residuals resulted from fitting $ln(\varepsilon_{exp}^{SL})$ versus $ln(E_{\gamma}/E_{0})$, being $E_{0}$ = 1 keV.  (*) In the case of the ^7^Be, the efficiency value for slotted filters was obtained by using the fitting function given in Table S.2. | | | | | | | | | | |
| --- | --- | --- | --- | --- | --- | --- | --- | --- | --- | --- |
| RN | $E_{\gamma}$ (keV) | $m_{RGU-1}^{SL (1)}$ (g) = 0.5115(2) | |  | $m_{RGU-1}^{SL (2)}$ (g) = 0.5014(2) | |  | $\varepsilon_{exp}^{SL}$ | $\sigma\left( \varepsilon_{exp}^{SL} \right)$ | *Res* (%) |
|  |  | $\varepsilon_{exp}^{SL (1)}$ | $\sigma\left( \varepsilon_{exp}^{SL \left( 1 \right)} \right)$ |  | $\varepsilon_{exp}^{SL (2)}$ | $\sigma\left( \varepsilon_{exp}^{SL \left( 2 \right)} \right)$ |  |  |  |  |
| ^210^Pb | 46.54 | 0.174 | 0.007 |  | 0.170 | 0.006 |  | 0.172 | 0.005 | 0.28 |
| ^226^Ra | 185.96 | 0.118 | 0.009 |  | 0.123 | 0.009 |  | 0.121 | 0.007 | 0.21 |
| ^214^Pb | 295.22 | 0.0697 | 0.0018 |  | 0.0732 | 0.0017 |  | 0.0715 | 0.0013 | 2.2 |
|  | 351.93 | 0.0601 | 0.0012 |  | 0.0625 | 0.0012 |  | 0.0613 | 0.0009 | -3.2 |
| ^214^Bi | 609.31 | 0.0297 | 0.0007 |  | 0.0306 | 0.0007 |  | 0.0302 | 0.0005 | 3.1 |
|  | 1120.29 | 0.0179 | 0.0010 |  | 0.0179 | 0.0009 |  | 0.0179 | 0.0007 | -1.7 |
|  | 1764.49 | 0.0139 | 0.0008 |  | 0.0144 | 0.0008 |  | 0.0141 | 0.0006 | 0.78 |
| ^7^Be | 477.60 |  |  |  |  |  |  | (*)0.0410 | 0.0010 |  |

| **Table S.2**  Parameters and their uncertainties, $a_{k}^{SL}$ and $\sigma\left( a_{k}^{SL} \right)$, respectively, being k = 0, 1, 2, 3, resulted from fitting the logarithm of the experimental efficiencies obtained in the case of the slotted filters (SL), $\varepsilon_{exp}^{\mathrm{SL}}$, versus $ln(E_{\gamma}/E_{0})$, being $E_{0}$ = 1 keV. | | | |
| --- | --- | --- | --- |
| Parameter | $a_{k}^{SL}$ | $\sigma\left( a_{k}^{SL} \right)$ | $R^{2}$ |
| $a_{3}^{SL}$ | 0.145 | 0.002 | 0.9995 |
| $a_{2}^{SL}$ | -2.58 | 0.04 |  |
| $a_{1}^{SL}$ | 14.2 | 0.2 |  |
| $a_{0}^{SL}$ | -26.3 | 0.4 |  |

| **Table S.3**  Experimental efficiencies obtained in the calibration procedure for circular filters whose diameter is 150 mm (C–150), $\varepsilon_{exp}^{C-150 (j)}$, where j = 1, 2, 3. Then, $m_{RGU-1}^{C-150 (j)}$ is the amount of RGU-1 added to each filter “j” in order to carry out the efficiency calibration. By using the $\varepsilon_{exp}^{C-150 (j)}$ values obtained for the three filters, an average experimental efficiency was calculated, $\varepsilon_{exp}^{C-150}$, for each $E_{\gamma}$, where $\sigma\left( \varepsilon_{exp}^{C-150 (j)} \right)$ and $\sigma\left( \varepsilon_{exp}^{C-150} \right)$ are the uncertainties related to $\varepsilon_{exp}^{C-150 (j)}$ and $\varepsilon_{exp}^{C-150}$, respectively, and *Res* (%) are the relative residuals resulted from fitting $ln(\varepsilon_{exp}^{C-150})$ versus $ln(E_{\gamma}/E_{0})$, being $E_{0}$ = 1 keV.  (*) In the case of the ^7^Be, the efficiency value for circular filters (Ø = 150 mm) was obtained by using the fitting function given in Table S.4. | | | | | | | | | | | | | |
| --- | --- | --- | --- | --- | --- | --- | --- | --- | --- | --- | --- | --- | --- |
| RN | $E_{\gamma}$ (keV) | $m_{RGU-1}^{C-150 (1)}$ (g) = 0.5029(2) | |  | $m_{RGU-1}^{C-150 (2)}$ (g) = 0.4898(2) | |  | $m_{RGU-1}^{C-150 (3)}$ (g) = 0.5006(2) | |  | $\varepsilon_{exp}^{C-150}$ | $\sigma\left( \varepsilon_{exp}^{C-150} \right)$ | *Res* (%) |
|  |  | $\varepsilon_{exp}^{C-150 (1)}$ | $\sigma\left( \varepsilon_{exp}^{C-150 \left( 1 \right)} \right)$ |  | $\varepsilon_{exp}^{C-150 (2)}$ | $\sigma\left( \varepsilon_{exp}^{C-150 \left( 2 \right)} \right)$ |  | $\varepsilon_{exp}^{C-150 (3)}$ | $\sigma\left( \varepsilon_{exp}^{C-150 \left( 3 \right)} \right)$ |  |  |  |  |
| ^210^Pb | 46.54 | 0.181 | 0.014 |  | 0.157 | 0.007 |  | 0.150 | 0.013 |  | 0.1619 | 0.007 | -2.3 |
| ^226^Ra | 185.96 | 0.093 | 0.012 |  | 0.114 | 0.010 |  | 0.128 | 0.014 |  | 0.111 | 0.007 | 0.17 |
| ^214^Pb | 295.22 | 0.070 | 0.003 |  | 0.0684 | 0.0019 |  | 0.076 | 0.004 |  | 0.0715 | 0.0018 | -4.1 |
|  | 351.93 | 0.0615 | 0.0023 |  | 0.0561 | 0.0013 |  | 0.0637 | 0.0023 |  | 0.0603 | 0.0012 | -7.3 |
| ^214^Bi | 609.31 | 0.0296 | 0.0014 |  | 0.0280 | 0.0008 |  | 0.0332 | 0.0015 |  | 0.0302 | 0.0007 | -2.3 |
|  | 1120.29 | 0.0146 | 0.0018 |  | 0.0172 | 0.0011 |  | 0.0180 | 0.0020 |  | 0.0165 | 0.0010 | -2.2 |
|  | 1764.49 | 0.0136 | 0.0017 |  | 0.0136 | 0.0009 |  | 0.0118 | 0.0016 |  | 0.0130 | 0.0008 | -5.7 |
| ^7^Be | 477.60 |  |  |  |  |  |  |  |  |  | (*)0.042 | 0.002 |  |

| **Table S.4**  Parameters and their uncertainties, $a_{k}^{C-150}$ and $\sigma\left( a_{k}^{C-150} \right)$, respectively, being k = 0, 1, 2, 3, resulted from fitting the logarithm of the experimental efficiencies obtained in the case of the circular filters whose diameter is 150 mm (C–150), $\varepsilon_{exp}^{C-150}$, versus $ln(E_{\gamma}/E_{0})$, being $E_{0}$ = 1 keV. | | | |
| --- | --- | --- | --- |
| Parameter | $a_{k}^{C-150}$ | $\sigma\left( a_{k}^{C-150} \right)$ | $R^{2}$ |
| $a_{3}^{C-150}$ | 0.128 | 0.004 | 0.9993 |
| $a_{2}^{C-150}$ | -2.31 | 0.08 |  |
| $a_{1}^{C-150}$ | 12.8 | 0.4 |  |
| $a_{0}^{C-150}$ | -24.0 | 0.8 |  |

| **Table S.5**  Experimental efficiencies obtained in the calibration procedure for circular filters whose diameter is 47 mm (C–47), $\varepsilon_{exp}^{C-47 (j)}$, where j = 1, 2, 3. Then, $m_{RGU-1}^{C-47 (j)}$ is the amount of RGU-1 added to each filter “j” in order to carry out the efficiency calibration. By using the $\varepsilon_{exp}^{C-47 (j)}$ values obtained for the three filters, an average experimental efficiency was calculated, $\varepsilon_{exp}^{C-47}$, for each $E_{\gamma}$, where $\sigma\left( \varepsilon_{exp}^{C-47 (j)} \right)$ and $\sigma\left( \varepsilon_{exp}^{C-47} \right)$ are the uncertainties related to $\varepsilon_{exp}^{C-47 (j)}$ and $\varepsilon_{exp}^{C-47}$, respectively, and *Res* (%) are the relative residuals resulted from fitting $ln(\varepsilon_{exp}^{C-47})$ versus $ln(E_{\gamma}/E_{0})$, being $E_{0}$ = 1 keV.  (*) In the case of the ^7^Be, the efficiency value for circular filters (Ø = 47 mm) was obtained by using the fitting function given in Table S.6. | | | | | | | | | | | | | |
| --- | --- | --- | --- | --- | --- | --- | --- | --- | --- | --- | --- | --- | --- |
| RN | $E_{\gamma}$ (keV) | $m_{RGU-1}^{C-47 (1)}$ (g) = 0.5029(2) | |  | $m_{RGU-1}^{C-47 (2)}$ (g) = 0.4898(2) | |  | $m_{RGU-1}^{C-47 (3)}$ (g) = 0.5006(2) | |  | $\varepsilon_{exp}^{C-47}$ | $\sigma\left( \varepsilon_{exp}^{C-47} \right)$ | *Res* (%) |
|  |  | $\varepsilon_{exp}^{C-47 (1)}$ | $\sigma\left( \varepsilon_{exp}^{C-47 \left( 1 \right)} \right)$ |  | $\varepsilon_{exp}^{C-47 (2)}$ | $\sigma\left( \varepsilon_{exp}^{C-47 \left( 2 \right)} \right)$ |  | $\varepsilon_{exp}^{C-47 (3)}$ | $\sigma\left( \varepsilon_{exp}^{C-47 \left( 3 \right)} \right)$ |  |  |  |  |
| ^210^Pb | 46.54 | 0.291 | 0.015 |  | 0.25 | 0.02 |  | 0.305 | 0.011 |  | 0.2818 | 0.0281 | -0.64 |
| ^226^Ra | 185.96 | 0.220 | 0.018 |  | 0.21 | 0.02 |  | 0.201 | 0.015 |  | 0.2096 | 0.0110 | 5.8 |
| ^214^Pb | 295.22 | 0.133 | 0.004 |  | 0.119 | 0.006 |  | 0.125 | 0.003 |  | 0.1254 | 0.0067 | -0.82 |
|  | 351.93 | 0.111 | 0.003 |  | 0.100 | 0.004 |  | 0.103 | 0.002 |  | 0.1045 | 0.0062 | -6.5 |
| ^214^Bi | 609.31 | 0.0505 | 0.0016 |  | 0.041 | 0.002 |  | 0.0456 | 0.0011 |  | 0.0458 | 0.0046 | 2.2 |
|  | 1120.29 | 0.023 | 0.002 |  | 0.023 | 0.003 |  | 0.0267 | 0.0015 |  | 0.0243 | 0.0021 | 5.0 |
|  | 1764.49 | 0.0228 | 0.0019 |  | 0.020 | 0.003 |  | 0.0240 | 0.0013 |  | 0.0223 | 0.0020 | -1.7 |
| ^7^Be | 477.60 |  |  |  |  |  |  |  |  |  | (*)0.064 | 0.002 |  |

| **Table S.6**  Parameters and their uncertainties, $a_{k}^{C-47}$ and $\sigma\left( a_{k}^{C-47} \right)$, respectively, being k = 0, 1, 2, 3, resulted from fitting the logarithm of the experimental efficiencies obtained in the case of the circular filters whose diameter is 47 mm (C–47), $\varepsilon_{exp}^{C-47}$, versus $ln(E_{\gamma}/E_{0})$, being $E_{0}$ = 1 keV. | | | |
| --- | --- | --- | --- |
| Parameter | $a_{k}^{C-47}$ | $\sigma\left( a_{k}^{C-47} \right)$ | $R^{2}$ |
| $a_{3}^{C-47}$ | 0.196 | 0.006 | 0.998 |
| $a_{2}^{C-47}$ | -3.47 | 0.10 |  |
| $a_{1}^{C-47}$ | 19.2 | 0.6 |  |
| $a_{0}^{C-47}$ | -34.7 | 1.0 |  |

| **Table S.7**  Experimental efficiencies obtained in the calibration procedure for rectangular filters (R), $\varepsilon_{exp}^{R (j)}$, where j = 1, 2, 3. Then, $m_{RGU-1}^{R (j)}$ is the amount of RGU-1 added to each filter “j” in order to carry out the efficiency calibration. By using the $\varepsilon_{exp}^{R (j)}$ values obtained for the three filters, an average experimental efficiency was calculated, $\varepsilon_{exp}^{R}$, for each $E_{\gamma}$, where $\sigma\left( \varepsilon_{exp}^{R (j)} \right)$ and $\sigma\left( \varepsilon_{exp}^{R} \right)$ are the uncertainties related to $\varepsilon_{exp}^{R (j)}$ and $\varepsilon_{exp}^{R}$, respectively, and *Res* (%) are the relative residuals resulted from fitting $ln(\varepsilon_{exp}^{R})$ versus $ln(E_{\gamma}/E_{0})$, being $E_{0}$ = 1 keV.  (*) In the case of the ^7^Be, the efficiency value for rectangular filters was obtained by using the fitting function given in Table S.8. | | | | | | | | | | | | | |
| --- | --- | --- | --- | --- | --- | --- | --- | --- | --- | --- | --- | --- | --- |
| RN | $E_{\gamma}$ (keV) | $m_{RGU-1}^{R (1)}$ (g) = 0.6018(2) | |  | $m_{RGU-1}^{R (2)}$ (g) = 0.5913(2) | |  | $m_{RGU-1}^{R (3)}$ (g) = 0.5914(2) | |  | $\varepsilon_{exp}^{R}$ | $\sigma\left( \varepsilon_{exp}^{R} \right)$ | *Res* (%) |
|  |  | $\varepsilon_{exp}^{R (1)}$ | $\sigma\left( \varepsilon_{exp}^{R \left( 1 \right)} \right)$ |  | $\varepsilon_{exp}^{R (2)}$ | $\sigma\left( \varepsilon_{exp}^{R \left( 2 \right)} \right)$ |  | $\varepsilon_{exp}^{R (3)}$ | $\sigma\left( \varepsilon_{exp}^{R \left( 3 \right)} \right)$ |  |  |  |  |
| ^210^Pb | 46.54 | 0.159 | 0.008 |  | 0.161 | 0.007 |  | 0.149 | 0.0085 |  | 0.1559 | 0.005 | -0.47 |
| ^226^Ra | 185.96 | 0.121 | 0.011 |  | 0.107 | 0.009 |  | 0.133 | 0.011 |  | 0.1196 | 0.006 | 2.9 |
| ^214^Pb | 295.22 | 0.0770 | 0.0025 |  | 0.0729 | 0.0020 |  | 0.0780 | 0.0024 |  | 0.0759 | 0.0013 | -0.85 |
|  | 351.93 | 0.0676 | 0.0017 |  | 0.0623 | 0.0014 |  | 0.0639 | 0.0016 |  | 0.0646 | 0.0009 | -5.3 |
| ^214^Bi | 609.31 | 0.0326 | 0.0010 |  | 0.0311 | 0.0008 |  | 0.0319 | 0.0010 |  | 0.0319 | 0.0005 | 0.42 |
|  | 1120.29 | 0.0172 | 0.0013 |  | 0.0170 | 0.0011 |  | 0.0186 | 0.0013 |  | 0.0176 | 0.0007 | 1.8 |
|  | 1764.49 | 0.0163 | 0.0012 |  | 0.0140 | 0.0009 |  | 0.0140 | 0.0011 |  | 0.0147 | 0.0006 | -2.6 |
| ^7^Be | 477.60 |  |  |  |  |  |  |  |  |  | (*)0.042 | 0.002 |  |

| **Table S.8**  Parameters and their uncertainties, $a_{k}^{R}$ and $\sigma\left( a_{k}^{R} \right)$, respectively, being k = 0, 1, 2, 3, resulted from fitting the logarithm of the experimental efficiencies obtained in the case of the rectangular filters (R), $\varepsilon_{exp}^{R}$, versus $ln(E_{\gamma}/E_{0})$, being $E_{0}$ = 1 keV. | | | |
| --- | --- | --- | --- |
| Parameter | $a_{k}^{R}$ | $\sigma\left( a_{k}^{R} \right)$ | $R^{2}$ |
| $a_{3}^{R}$ | 0.150 | 0.003 | 0.9991 |
| $a_{2}^{R}$ | -2.70 | 0.05 |  |
| $a_{1}^{R}$ | 15.0 | 0.3 |  |
| $a_{0}^{R}$ | -28.1 | 0.5 |  |

| **Table S.9**  Experimental efficiencies obtained in the calibration procedure for squared filters (SQ), $\varepsilon_{exp}^{SQ (j)}$, where j = 1, 2, 3. Then, $m_{RGU-1}^{SQ (j)}$, $m_{RGTh-1}^{SQ (j)}$ and $m_{RGK-1}^{SQ (j)}$ are the amounts of RGU-1, RGTh-1 and RGK-1, respectively, added to each filter “j” in order to carry out the efficiency calibration. By using the $\varepsilon_{exp}^{SQ (j)}$ values obtained for the three filters, an average experimental efficiency was calculated, $\varepsilon_{exp}^{SQ}$, for each $E_{\gamma}$, where $\sigma\left( \varepsilon_{exp}^{SQ (j)} \right)$ and $\sigma\left( \varepsilon_{exp}^{SQ} \right)$ are the uncertainties related to $\varepsilon_{exp}^{SQ (j)}$ and $\varepsilon_{exp}^{SQ}$, respectively, and *Res* (%) are the relative residuals resulted from fitting $ln(\varepsilon_{exp}^{SQ})$ versus $ln(E_{\gamma}/E_{0})$, being $E_{0}$ = 1 keV.  (*) In the case of the ^7^Be, the efficiency value for squared filters was obtained by using the fitting function given in Table S.10. | | | | | | | | | | | | | |
| --- | --- | --- | --- | --- | --- | --- | --- | --- | --- | --- | --- | --- | --- |
| RN | $E_{\gamma}$ (keV) | $m_{RGU-1}^{SQ (1)}$, $m_{RGTh-1}^{SQ (1)}$, $m_{RGK-1}^{SQ (1)}$ = 0.8337(2) g | |  | $m_{RGU-1}^{SQ (2)}$, $m_{RGTh-1}^{SQ (2)}$, $m_{RGK-1}^{SQ (2)}$ = 0.8339(2) g | |  | $m_{RGU-1}^{SQ (3)}$, $m_{RGTh-1}^{SQ (3)}$, $m_{RGK-1}^{SQ (3)}$ = 0.8302(2) g | |  | $\varepsilon_{exp}^{SQ}$ | $\sigma\left( \varepsilon_{exp}^{SQ} \right)$ | *Res* (%) |
|  |  | $\varepsilon_{exp}^{SQ (1)}$ | $\sigma\left( \varepsilon_{exp}^{SQ \left( 1 \right)} \right)$ |  | $\varepsilon_{exp}^{SQ (2)}$ | $\sigma\left( \varepsilon_{exp}^{SQ \left( 2 \right)} \right)$ |  | $\varepsilon_{exp}^{SQ (3)}$ | $\sigma\left( \varepsilon_{exp}^{SQ \left( 3 \right)} \right)$ |  |  |  |  |
| ^210^Pb | 46.54 | 0.085 | 0.005 |  | 0.094 | 0.005 |  | 0.099 | 0.005 |  | 0.092 | 0.007 | -0.60 |
| ^234^Th | 63.29 | 0.103 | 0.011 |  | 0.123 | 0.012 |  | 0.106 | 0.011 |  | 0.112 | 0.011 | 1.4 |
| ^226^Ra | 185.96 | 0.081 | 0.007 |  | 0.090 | 0.007 |  | 0.080 | 0.007 |  | 0.083 | 0.006 | 0.026 |
| ^212^Pb | 238.63 | 0.068 | 0.002 |  | 0.075 | 0.002 |  | 0.069 | 0.002 |  | 0.067 | 0.004 | -4.9 |
| ^214^Pb | 295.22 | 0.0472 | 0.0012 |  | 0.0564 | 0.0013 |  | 0.0582 | 0.0014 |  | 0.055 | 0.006 | 1.7 |
|  | 351.93 | 0.0420 | 0.0008 |  | 0.0475 | 0.0009 |  | 0.0478 | 0.0009 |  | 0.046 | 0.003 | -0.21 |
| ^208^Tl | 583.19 | 0.0253 | 0.0011 |  | 0.0270 | 0.0012 |  | 0.0240 | 0.0011 |  | 0.0268 | 0.0016 | 5.3 |
| ^214^Bi | 609.31 | 0.0226 | 0.0005 |  | 0.0250 | 0.0005 |  | 0.0257 | 0.0006 |  | 0.0256 | 0.0017 | 5.0 |
|  | 1120.29 | 0.0142 | 0.0007 |  | 0.0166 | 0.0007 |  | 0.0144 | 0.0008 |  | 0.0151 | 0.0013 | 0.22 |
|  | 1764.49 | 0.0114 | 0.0006 |  | 0.0132 | 0.0007 |  | 0.0116 | 0.0007 |  | 0.0121 | 0.0007 | 1.7 |
| ^228^Ac | 911.16 | 0.0185 | 0.0010 |  | 0.0203 | 0.0011 |  | 0.0194 | 0.0011 |  | 0.0176 | 0.0008 | -9.2 |
| ^40^K | 1460.83 | 0.0132 | 0.0007 |  | 0.0132 | 0.0007 |  | 0.0123 | 0.0007 |  | 0.0129 | 0.0006 | 0.22 |
| ^7^Be | 477.60 |  |  |  |  |  |  |  |  |  | (*)0.0330 | 0.0013 |  |

| **Table S.10**  Parameters and their uncertainties, $a_{k}^{SQ}$ and $\sigma\left( a_{k}^{SQ} \right)$, respectively, being k = 0, 1, 2, 3, resulted from fitting the logarithm of the experimental efficiencies obtained in the case of the squared filters (SQ), $\varepsilon_{exp}^{SQ}$, versus $ln(E_{\gamma}/E_{0})$, being $E_{0}$ = 1 keV. | | | |
| --- | --- | --- | --- |
| Parameter | $a_{k}^{SQ}$ | $\sigma\left( a_{k}^{SQ} \right)$ | $R^{2}$ |
| $a_{3}^{SQ}$ | 0.134 | 0.007 | 0.998 |
| $a_{2}^{SQ}$ | -2.43 | 0.12 |  |
| $a_{1}^{SQ}$ | 13.7 | 0.7 |  |
| $a_{0}^{SQ}$ | -26.6 | 1.3 |  |
